# Solitonic Josephson-based meminductive systems

**DOI:** 10.1038/srep46736

**Published:** 2017-04-24

**Authors:** Claudio Guarcello, Paolo Solinas, Massimiliano Di Ventra, Francesco Giazotto

**Affiliations:** 1 SPIN-CNR, Via Dodecaneso 33, I-16146 Genova, Italy; 2 NEST, Istituto Nanoscienze-CNR and Scuola Normale Superiore, Piazza S. Silvestro 12, I-56127 Pisa, Italy; 3 Radiophysics Department, Lobachevsky State University, Gagarin Ave. 23, 603950 Nizhny Novgorod, Russia; 4 Department of Physics, University of California, San Diego, La Jolla, California 92093, USA

## Abstract

Memristors, memcapacitors, and meminductors represent an innovative generation of circuit elements whose properties depend on the state and history of the system. The hysteretic behavior of one of their constituent variables, is their distinctive fingerprint. This feature endows them with the ability to store and process information on the same physical location, a property that is expected to benefit many applications ranging from unconventional computing to adaptive electronics to robotics. Therefore, it is important to find appropriate memory elements that combine a wide range of memory states, long memory retention times, and protection against unavoidable noise. Although several physical systems belong to the general class of memelements, few of them combine these important physical features in a single component. Here, we demonstrate theoretically a superconducting memory based on solitonic long Josephson junctions. Moreover, since solitons are at the core of its operation, this system provides an intrinsic topological protection against external perturbations. We show that the Josephson critical current behaves hysteretically as an external magnetic field is properly swept. Accordingly, long Josephson junctions can be used as multi-state memories, with a controllable number of available states, and in other emerging areas such as memcomputing, i.e., computing directly in/by the memory.

Circuit elements, specifically, resistors, capacitors, and inductors with memory^1^,^2^,^3^,^4^,^5^,^6^,^7^,^8^, i.e., elements with characteristics that depend on the past states through which the system has evolved, have recently received increasing attention. Beyond the obvious applications in storing information, these elements can be combined in complex circuits to perform logic^9^ and unconventional computing operations^10^,^11^,^12^,^13^,^14^,^15^,^16^ in massive parallel schemes^17^, and in the same physical location where storing occurs. Superconducting circuits that store and manipulate information are particularly appealing in view of their low-energy operation. Among these, a superconducting tunnel junction-based memristor was recently suggested^18^,^19^,^20^. However, this type of element does not feature controllable multiple states that can be easily protected against unavoidable noise, due to a stochastic drift of the memory^15^.

Our proposal instead is based on a *long* rectangular tunnel Josephson junction (LJJ) subject to a suitable periodical driving. A tunnel Josephson junction is a quantum device formed by sandwiching a thin insulating layer between two superconducting electrodes, and “long” refers to the physical length of the junction (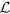) which is supposed to exceed the Josephson penetration depth (*λ*_*J*_). A scheme of a LJJ with an in-plane magnetic field (*H*_*ext*_) is shown in Fig. 1a. A LJJ is the prototypical system to investigate solitons^21^,^22^ in a fully solid-state environment, and the history-dependent behavior that we envision stems from how solitons rearrange their configuration along the junction under the effect of an external magnetic field^23^,^24^.

## Results and Discussion

The phase dynamics of a LJJ is described by the sine-Gordon equation^25^,^26^,^27^,^28^:


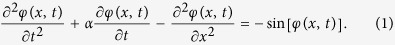


Above, *φ* is the macroscopic quantum phase difference between the superconductors, *α* denotes the intensity of the damping effect, *x* is the spatial coordinate along the junction, and *t* is the time (see SI). The boundary conditions of equation (1) read


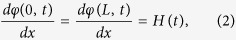


where *H*(*t*) is the normalized time-dependent external magnetic field, and 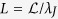 is the normalized length of the junction. By varying *H*(*t*), the phase *φ* evolves according to equations (1) and (2). For a spatially homogeneous supercurrent density, the Josephson critical current 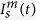 of the junction shows a “Fraunhofer-like” diffraction pattern consisting of overlapping lobes as the magnetic field is increased, and described by the following equation^29^,^30^,^31^:


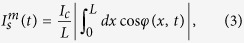


where *I*_*c*_ is the zero-field, zero-temperature junction critical current. This behavior is shown in Fig. 2a as the driving magnetic field is swept “forward” from zero. A diffraction lobe corresponds to a specific number of solitons present along the junction^23^,^31^. When the external magnetic field penetrates the junction edges it induces Josephson vortices along the weak-link, according to the nonlinearity of equation (1). These vortices, i.e., solitons, are induced by persistent supercurrent loops carrying a quantum of magnetic flux, Φ_0_^21^,^22^. The critical current, and the resulting patterns as the driving field is swept, are the physical quantities on which we focus since they can be measured with conventional techniques. In all forthcoming calculations we use parameters typical of Nb/AlOx/Nb tunnel junctions as the ideal materials combination to implement solitonic Josephson-based meminductive structures.

Figure 2b shows the diffraction pattern of the critical current when the magnetic field direction is reversed. The resulting “backward” diffraction pattern markedly differs from the forward pattern shown in Fig. 2a. For a given magnetic field *H*, the current state in which the system is found depends on the field history. This is a remarkable feature of the dissipative solitonic dynamics described by equation (1). Different current states correspond to different numbers of solitons arranged along the junction, and the transition from a diffraction lobe to another corresponds to the injection, or the ejection, of solitons^31^. As in any dissipative dynamics, the state of the system is not only determined by the value of the drive but it also depends on the path followed by the system. This induces the forward-backward asymmetry, and the hysteretic diffraction patterns shown in Fig. 2a,b. In the forward pattern, the first lobe corresponds to the Meissner state, i.e., zero solitons in the junction, whereas by exceeding a threshold value 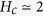 the second lobe begins and solitons in the form of magnetic fluxons penetrate into the junction. This value of the critical field characterizes the diffraction patterns of the Josephson critical current in both overlap and inline LJJs^26^,^32^,^33^. For *H* > 0, the backward dynamics is strictly described by *N*-solitons solutions, with *N* ≥ 1. The amount of solitons exited depends on both the field intensity and the length of the junction.

Figure 2b–d display the forward-backward diffraction patterns as a function of the junction length. Specifically, by increasing the length, the number of lobes forming the pattern grows, and the hysteretic asymmetry between forward and backward patterns is enhanced. Notably, *L* can be tuned as well by changing the junction operation temperature (*T*) owing to the temperature dependence of *λ*_*J*_ (*T*).

The presence of both the hysteretic behavior of the critical current and highly-distinguishable current states suggests possible applications of the LJJ. For instance, this device can be used as a field-controlled memelement^1^,^5^,^7^, in which the time-dependent input/output related variables are the external magnetic field *H (t*) and the Josephson critical current 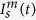, respectively. We envisage here a memelement with distinct memory states which make use of the lobes of the forward/backward diffraction patterns. For a given applied magnetic field, the memelement state is determined by the value of the critical current, the latter keeping track of the field history, and pointing to a specific number of solitons present in the junction. Since the critical supercurrent and the magnetic field are the variables yielding the history-dependent behavior, our junction can be regarded as a *meminductive system*^5^,^7^,^34^,^35^,^36^,^37^, specifically, a field-controlled solitonic Josephson-based meminductive system (SJMS).

**Figure 1:**
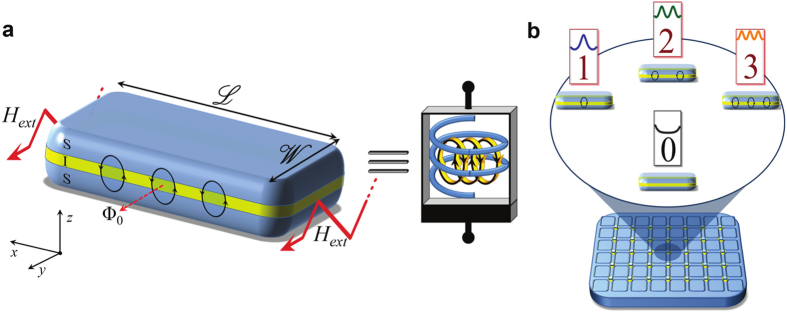
Solitonic Josephson-based meminductive system. (**a**) A superconductor-insulator-superconductor (SIS) rectangular long Josephson junction (LJJ) excited by an homogeneous external periodical magnetic field *H*_*ext*_. Here, we refer to the normalized field *H* in place of *H*_*ext*_ (see SI). The length and the width of the junction are 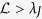 and 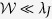, respectively, where *λ*_*J*_ is the Josephson penetration depth. A LJJ excited by a magnetic flux falls into the category of field-controlled meminductive systems, since the input and output variables are the applied magnetic field and the Josephson critical current, respectively. The symbol used to represent the *solitonic* Josephson-based meminductive system (SJMS) is shown. Fluxons (Φ_0_) within the junction surrounded by supercurrent loops are also represented. (**b**) Schematic of a possible memory drive formed by an ensemble of SJMSs. The core of the device is a LJJ excited by an in-plane magnetic field, with specific read-out electronics for the critical current. As an example, we display here a junction with length 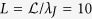 by which a 4-state memory element can be defined. These distinct states are labelled by the number of solitons arranged along the junction. The peaks in the *dφ*/*dx* curves (see SI) and the number of loops of Josephson current surrounding the fluxons are indicated as well.

**Figure 2:**
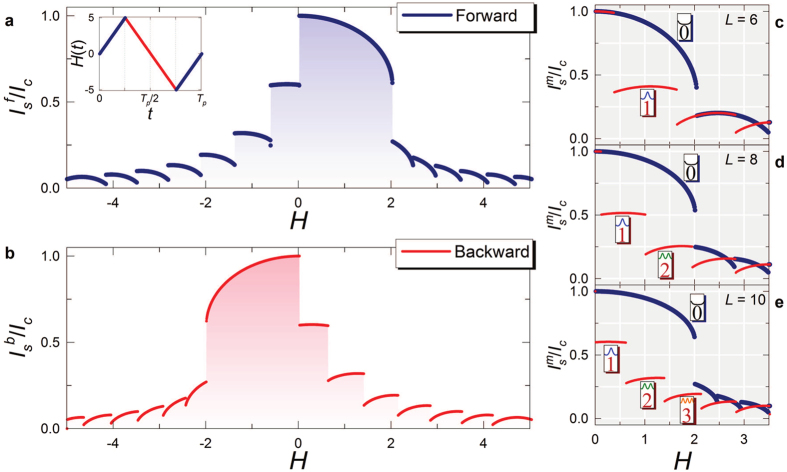
Josephson critical current diffraction patterns. (**a**,**b)**, Normalized Josephson critical currents 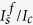 and 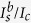 as the driving field *H* is swept forward from *H* = 0 to *H* = 5 (right half of panel a), then backward from *H* = 5 to *H* = −5 (panel b) and again forward from *H* = −5 to *H* = 0 (left half of panel (a). The inset in panel (a) shows one period (*T*_*H*_) of the driving field. The critical current as a function of *H*(*t*) exhibits a diffraction-like pattern formed by lobes which are directly related to the number of solitons arranged along the junction. By sweeping the magnetic field forward and then backward leads to the appearance of a clear hysteretic behavior. This is a distinctive signature of any memdevice. According to this hysteretic behavior, the Josephson junction can be effectively used as a multi-state memory. For any specific range of magnetic field values, each state of the memory is represented by a forward or backward diffraction lobe, labeled by the number of excited solitons present along the junction. (**c**,**d** and **e**), Diffraction patterns for a few junction lengths *L*. The number of memory states provided by the SJMS can be changed by varying the junction length. The memory states associated with current lobes are indicated with the same notation as in Fig. 1b.

More generally, the LJJ can be thought as a multi-state memory in which each memory state is represented by a specific diffraction lobe, and labeled by the number of excited solitons (see Fig. 1b). For example, by referring to the diffraction patterns shown in Fig. 2a,b, three backward lobes can be easily recognized within the range *H* ∈ [0, 2] in clear contrast to one single forward lobe, by which a 4-state memory could be built.

**Figure 3:**
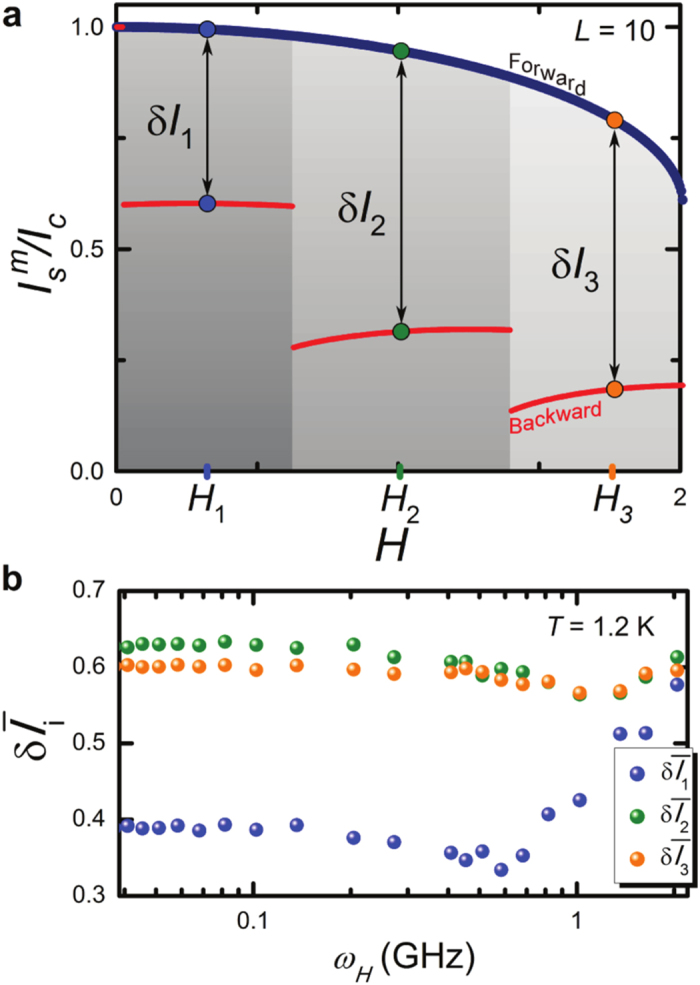
Frequency response of the memory states. (**a**) Forward and backward diffraction patterns for 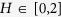 and *L* = 10. For each backward diffraction lobe, we have considered the middle magnetic field value *H*_*i*_, and calculated the current difference 
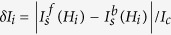
 (*i* = 1, 2, 3), where 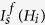 and 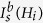 are the corresponding forward and backward critical currents. (**b)** Difference 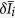 (*i* = 1, 2, 3) between average forward and backward diffraction patterns 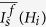 and 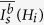, computed by averaging over *N*_*exp*_ = 100 numerical realizations of the Josephson critical current, as a function of the driving frequency *ω*_*H*_ for *T* = 1.2 K. The memory states are stable up to *ω*_*H*_ ~ 0.5 GHz. At higher frequencies, i.e., 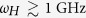, the system is no more able to respond to the fast driving.

On general grounds, a good memelement has to read/write in short times, and has to be sufficiently robust against external fluctuations (noise) that tend to destroy the stored information. On the one hand, reading the state of the SJMS can be performed by conventional well-established techniques, or via a Josephson sensor^38^, based on the variations of the kinetic inductance of a junction working in the dissipationless regime inductively coupled with a SQUID, or even by an interferometer reading the magnetic flux variations through the JJ. On the other hand, the writing process of each memory state depends on the operating frequency (*ω*_*H*_) of the magnetic field, and on the ability of the system to follow a fast periodic driving. To quantify the LJJ memdevice performance as the driving frequency and the temperature are changed we make use of a figure of merit defined by the difference between the forward and backward critical currents, 
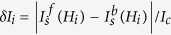
, where *H*_*i*_ is the magnetic field at the midpoint of the *i*-th backward diffraction lobe, as shown in Fig. 3a for *i* = 1, 2, 3. For large *δI*_*i*_ one can safely distinguish distinct memory states, namely, the current states. Furthermore, to further characterize our memdevice we have included a Gaussian thermal fluctuation term in equation (1) (see SI) thereby making the SJMS a *stochastic* memory element^7^ whereas a noiseless driving field source was considered. The relevant supercurrent differences (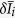) are then calculated between the averaged diffraction patterns. Recently, the effects of the noise on the performance of several memory devices has been investigated^15^,^18^,^39^,^40^,^41^,^42^,^43^,^44^,^45^.

Independently of the physical mechanism defining the state of the device, the memelement response is usually strongly dependent on the frequency of the input drive^10^,^46^,^47^. At low frequencies, the system has enough time to adjust its state to the instant value of the drive, so that the device non-linearly behaves and a hysteretic evolution results. Conversely, at high frequencies, there is not enough time for any change during an oscillation period of the drive.

Figure 3b shows 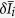 as a function of the driving frequency *ω*_*H*_, for *T* = 1.2 K. The memory states defined in Fig. 3a are stable up to a driving frequency *ω*_*H*_ ~ 0.5 GHz. At higher frequencies, i.e., for 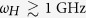, the system is not able to respond anymore to the fast driving. In this region of frequencies, 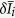 tends to increase (see SI), the diffraction patterns are not stable, and therefore cannot be used to safely distinguish the memory states.

As expected, due to its topological nature the LJJ memory shows remarkable robustness against thermal disturbances: being a soliton-based memelement, it is intrinsically protected against small fluctuations. Indeed, the states of the memory are associated to the number of solitons present in the LJJ and, therefore, are quantized^31^. The creation of a soliton is a macroscopic quantum phenomenon involving crossing of a potential barrier^31^. Far away from the superconducting critical temperature (*T*_*c*_), the presence of an energy barrier in a damped dynamics prevents noise-induced state degradations, i.e., the so-called “stochastic catastrophe”^15^.

**Figure 4:**
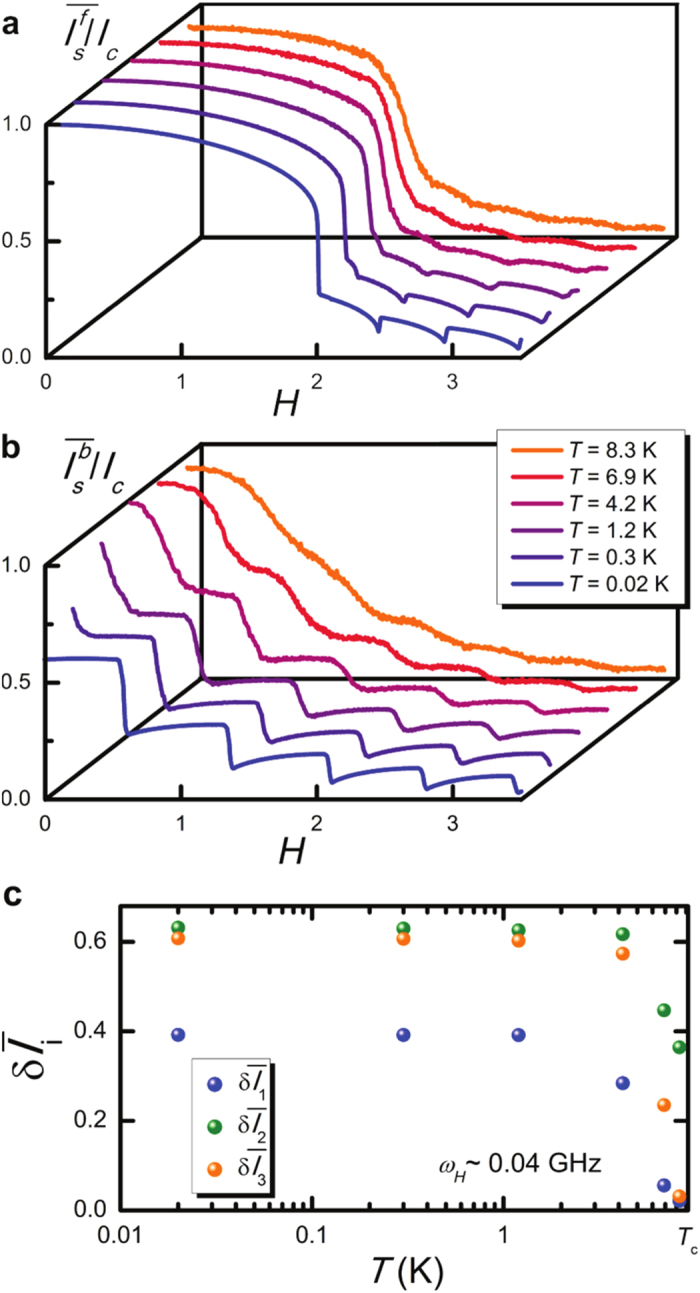
Effects of the temperature. (**a**,**b**) Average forward and backward diffraction patterns 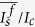 and 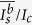, respectively, calculated for a few temperatures, *L* = 10, and *ω*_*H*_ ~ 0.04 GHz. The patterns are computed by averaging over *N*_*exp*_ = 100 numerical realizations of the critical current as the magnetic field is swept forward and backward when thermal fluctuations are taken into account. The legend in panel (b) refers to both panels. (c) Differences 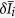 (*i* = 1, 2, 3) for *L* = 10 and *ω*_*H*_ ~ 0.04 GHz calculated in correspondence of the temperatures set to obtain the results shown in panels (a,b). By approaching the superconducting critical temperature (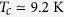 for a Nb/AlOx/Nb JJ) the forward and backward diffraction patterns tend to superimpose, and 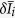 vanishes.

Figure 4 emphasizes the robustness of the SJMS against thermal fluctuations, as the driving frequency is set to *ω*_*H*_ ~ 0.04 GHz. Specifically, here we show how the temperature affects the forward (Fig. 4a) and backward (Fig. 4b) diffraction patterns. In particular, by increasing the temperature leads to a smoothing of the interference patterns with broadened transitions between lobes due to noise-induced creation or destruction of solitons. Nevertheless, the memory states tend to degrade only for somewhat high temperatures approaching *T*_*c*_ (see the results for T > 4.2 K in Fig. 4a,b).

Finally, the stability of our Josephson-based memory as the temperature is changed is quantified in Fig. 4c. In particular, the memory states turn out to be stable against large temperature variations, i.e., 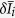 is roughly constant as long as 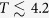 K. For higher temperatures, the average forward/backward diffraction patterns tend to superimpose so that 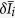 vanishes with the following suppression of the memory states at the critical temperature.

## Conclusion

In summary, we have suggested long Josephson junctions excited by an external magnetic field as prototypical multi-state superconducting memories. Our proposal for a memory element is based on the characteristic hysteretic behavior of the critical supercurrent as the driving field is swept. The resulting memelement realizes a multi-state memory with a number of states controllable via the effective length of the junction. The solitonic nature at the origin of the critical current hysteresis makes these memory states stable and robust against thermal fluctuations. Our memory scheme represents the first endeavor to combine superconductivity and solitons physics in one single memelement, and could find potential application in various emerging areas such as logic in memory and unconventional computing^16^,^17^.

## Methods

### Computational Details

The phase dynamics and the behaviors of the Josephson critical current presented in this work were calculated numerically by using a Fortran computer program.

To give a realistic estimate of the physical quantities used in the computations, both the superconductors and the insulator making the junction have to be chosen. Therefore, let us set a Nb/AlO/Nb junction, characterized by a resistance per area *R*_*a*_ = 50 Ω*μ*m^2^ and a specific capacitance *C*_*s*_ = 50*fF*/*μm*^2^. Moreover, a length-to-Josephson-penetration-depth ratio equal to *L* = 10 is considered.

At low temperatures, the critical current reads^26^ 
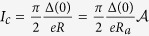
, where Δ(*T*) is the BCS superconducting energy gap and 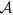 is the junction area. Accordingly, 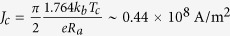, *T*_c_ = 9.2 K being the Nb critical temperature. The effective magnetic thickness is equal to 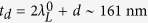, the London penetration depth of a Nb thin film being 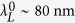 and setting *d* = 1 nm. Through the Josephson penetration depth 
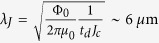
, the linear dimensions 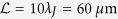 and 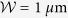 and the area 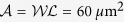 of the junction can be set. Therefore, the capacitance and the resistance of the device can be estimated, 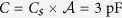 and 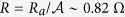, respectively. Consequently, the plasma frequency and the damping parameter read 
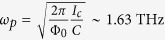
 and 
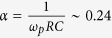
, respectively. The driving external field is equal to 
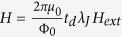
, so that 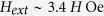.

Some of these quantities have an explicit dependence on the temperature. In particular, for identical superconductors^26^, the effective magnetic thickness *t*_*d*_(*T*) depends on *T* through the London penetration depth 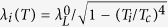, and the Josephson critical current *I*_*c*_(*T*) depends on *T* through the Ambegaokar and Baratoff formula^26^. Accordingly, the values used in the numerical calculations for the plasma frequency *ω*_*p*_(*T*), the damping parameter *α*(*T*), the Josephson penetration depth *λ*_*J*_(*T*), and the normalized length 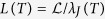 are adjusted by changing the temperature.

Concerning the normalized length, if, for instance, *L*(*T* → 0) = 10, as the temperature is increased to T* = 0.8 *T*_*c*_ the corresponding normalized length becomes 
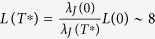
.

## Additional Information

**How to cite this article**: Guarcello, C. *et al*. Solitonic Josephson-based meminductive systems. *Sci. Rep.* **7**, 46736; doi: 10.1038/srep46736 (2017).

**Publisher's note:** Springer Nature remains neutral with regard to jurisdictional claims in published maps and institutional affiliations.

## Supplementary Material

Supplementary Information
