# Supplementary material for: Solitonic Josephson-based meminductive systems
Source: Sci Rep. 2017 Apr 24;7:46736. doi: 10.1038/srep46736 (PMC5402261; doi:10.1038/srep46736)
Supplement: Supplementary Information [file srep46736-s1.pdf]

Solitonic Josephson-based meminductive systems
Supplementary Information

Claudio Guarcello*[1,2,3], Paolo Solinas†[1], Massimiliano Di Ventra‡[4], and Francesco Giazotto§[2]

[1]SPIN-CNR, Via Dodecaneso 33, 16146 Genova, Italy
[2]NEST, Istituto Nanoscienze-CNR and Scuola Normale Superiore, Piazza S. Silvestro 12, I-56127 Pisa, Italy
[3]Radiophysics Department, Lobachevsky State University, Gagarin Ave. 23, 603950 Nizhny Novgorod, Russia
[4]Department of Physics, University of California, San Diego, La Jolla, California 92093, USA

February 7, 2017

Contents

1 The sine-Gordon equation and its solutions **S2**

2 The critical current diffraction patterns **S3**

3 Thermal effects **S5**

4 The frequency response **S7**

5 Memory devices and the response function **S9**

*e-mail: claudio.guarcello@nano.cnr.it
†e-mail: paolo.solinas@spin.cnr.it
‡e-mail: diventra@physics.ucsd.edu
§e-mail: francesco.giazotto@sns.it

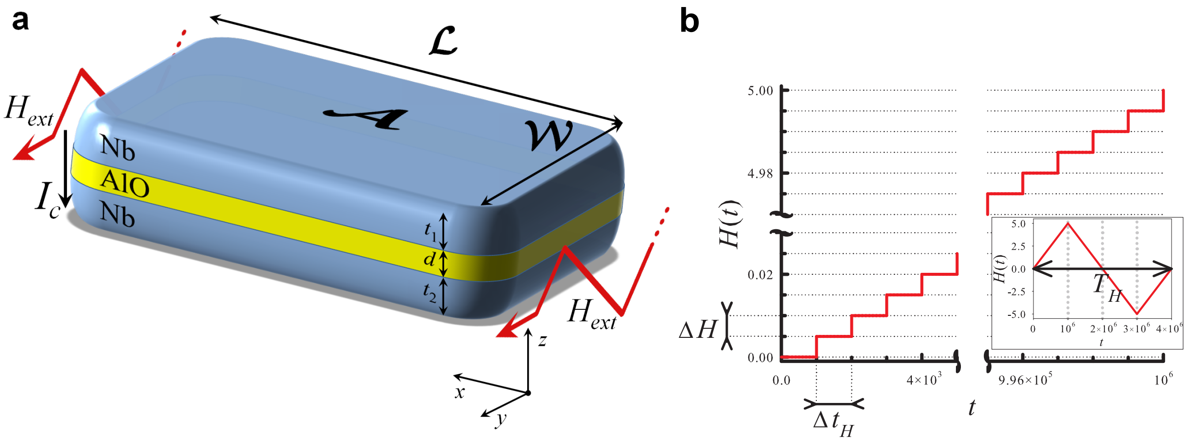

Figure 1: **a**, A Nb/AlO/Nb long Josephson junction (LJJ) in the presence of a homogeneous external magnetic field H_{ext} applied in the y direction. The length and the width of the junction are $\mathcal{L} > \lambda_{_J}$ and $\mathcal{W} \ll \lambda_{_J}$ (according to the long junction regime), respectively, and $\mathcal{A} = \mathcal{L} \times \mathcal{W}$ is the junction area, $\lambda_{_J}$ being the Josephson penetration depth. Moreover, t_i and d denote the thicknesses of the i-th superconductor and the insulating interlayer, respectively. **b**, Numerical implementaion of the magnetic field drive. Normalized staircase external magnetic field $H(t)$, formed by small steps with height $\Delta H = 0.005$ kept constant for time intervals $\Delta t_H = 10^3$, with $H_{max} = 5$. In the inset, a driving period $T_H = 4 \times 10^6$ of $H(t)$. Times are normalized with respect to the Josephson plasma frequency.

1 The sine-Gordon equation and its solutions

In Fig. 1(a), a long and narrow Nb/AlO/Nb Josephson junction (JJ) is represented. The electrodynamics of a long JJ is usually described by a partial differential equation for the order parameter φ, namely the phase difference between the wavefunctions describing the carriers in the superconducting electrodes. In normalized units, the perturbed sine-Gordon (SG) equation reads [1, 2, 3, 4, 5]

$$\frac{\partial^2\varphi}{\partial t^2} + \alpha\frac{\partial\varphi}{\partial t} - \frac{\partial^2\varphi}{\partial x^2} = -\sin(\varphi), \qquad (1)$$

with boundary conditions taking into account the normalized external magnetic field $H(t)$

$$\frac{d\varphi(0,t)}{dx} = \frac{d\varphi(L,t)}{dx} = H(t). \qquad (2)$$

In equation (1), space and time variables are normalized to the *Josephson penetration depth* $\lambda_{_J}$ and the *Josephson plasma frequency* ω_p, respectively. They read [2]

$$\lambda_{_J} = \sqrt{\frac{\Phi_0}{2\pi\mu_0}\frac{1}{t_d J_c}} \qquad (3)$$

$$\omega_p = \sqrt{\frac{2\pi}{\Phi_0}\frac{I_c}{C}}, \qquad (4)$$

where R and C are the total resistance and capacitance of the JJ, $\Phi_0 = h/2e \simeq 2.067 \times 10^{-15}$Wb is the magnetic flux quantum, μ_0 is the vacuum permeability, I_c and $J_c = I_c/\mathcal{A}$ are the critical current and the critical current area density ($\mathcal{A}$ being the junction area). Moreover, $t_d = \lambda_1 + \lambda_2 + d$ is the effective magnetic thickness, λ_i being the London penetration depth of the superconductor S_i and d the interlayer thickness. If λ_i exceeds the thickness t_i of the i-th superconductor, the effective magnetic thickness has to be replaced by $\tilde{t}_d = \lambda_1 \tanh(t_1/2\lambda_1) + \lambda_2 \tanh(t_2/2\lambda_2) + d$. The magnetic field H_{ext} lies parallel to a symmetry axes of the junction and along y.

The Josephson penetration depth represents the length scale of the system, so that a JJ is regarded as long and narrow if the length and the width of the junction are $\mathcal{L} > \lambda_{_J}$ and $\mathcal{W} \ll \lambda_{_J}$, respectively.

In normalized unit, the linear dimensions of the junction read $L = \mathcal{L}/\lambda_J > 1$ and $W = \mathcal{W}/\lambda_J \ll 1$. Moreover in equation (1), $\alpha = (\omega_p RC)^{-1}$ is the damping parameter.

The SG equation admits traveling wave solutions, called *solitons* [6]. In the SG framework, a soliton is often referred to as a kink. For the unperturbed SG equation, i.e., $\alpha = 0$ in equation (1), solitons have the simple analytical expression [2]

$$\varphi(x-ut) = 4\arctan\left\{\exp\left[\pm\frac{(x-ut)}{\sqrt{1-u^2}}\right]\right\}, \tag{5}$$

where the sign $\pm$ is the polarity of the soliton (specifically, the minus sign defines an *antisoliton*) and u is the Swihart's velocity [2], namely, the largest group propagation velocity of the linear electromagnetic waves in long junctions. Specifically, the phase of a soliton (antisoliton) twists from 0 to 2π (from 2π to 0). Alternatively, $\varphi/2\pi$ has a "topological charge" $+1$ for each soliton and -1 for each antisoliton. Moreover, a SG soliton has a well defined physical meaning in the long JJ framework, since it carries a quantum of magnetic flux, Φ_0, induced by a supercurrent loop surrounding it, with the local magnetic field perpendicularly oriented with respect to the junction length [7]. Thus, a soliton is usually referred to as a *fluxon*, or a Josephson vortex, in the context of long JJ.

In equation (2), the normalized external magnetic field is $H(t) = \frac{2\pi\mu_0}{\Phi_0} t_d \lambda_J H_{ext}(t)$, where $H_{ext}(t)$ is the non-normalized external magnetic field, see Fig. 1a. For the numerical simulation, we have modeled $H(t)$ as a staircase function formed by steps with "treads" deep Δt_H and "risers" high ΔH [see Fig. 1(b)]. To perform a double-swept drive, $H(t)$ is first ramped up from zero to H_{max}, then reduced to $-H_{max}$ and subsequently raised again to zero. Accordingly, the driving period $T_H = 4(H_{max}/\Delta H)\Delta t_H$ and frequency $\omega_H^* = 1/T_H$ are defined.

2 The critical current diffraction patterns

The φ-dependent supercurrent as a function of the external magnetic field H can be expressed as

$$I_s(H) = \iint dxdy J_s(x,y) = \iint dxdy J_c(x,y)\sin[\varphi(x,y)], \tag{6}$$

where $J_s(x,y)$ is the supercurrent density per unit area and $J_c(x,y)$ is the Josephson critical current density. We denote with $i_c(x)$ the $J_c(x,y)$ integral in the direction of the magnetic field

$$i_c(x) = \int_{-\mathcal{W}/2}^{\mathcal{W}/2} J_c(x,y)dy, \tag{7}$$

so that the Josephson current becomes

$$I_s(H) = \int_{-\mathcal{L}/2}^{\mathcal{L}/2} i_c(x)\sin\varphi(x)dx. \tag{8}$$

In equation (8), $\varphi(x)$ is the phase difference induced by the applied magnetic field H_{ext}. In fact, φ depends on the local magnetic field $H_y(x)$ through the equations [2]

$$\frac{\partial\varphi}{\partial x} = \frac{2\pi\mu_0 t_d}{\Phi_0} H_y(x) = h_y(x) \qquad\qquad \frac{\partial\varphi}{\partial y} = 0. \tag{9}$$

The latter equation comes from the condition $\mathcal{W} \ll \lambda_J$, so that $\varphi(x,y) \equiv \varphi(x)$.

For a *short* rectangular JJ ($\mathcal{L} \ll \lambda_J$ and $\mathcal{W} \ll \lambda_J$) the external magnetic field fully penetrates the junction and is spatially homogeneous along it, namely $H_y(x) \equiv H_{ext}$, so that, according to equation (9), the phase is just linearly increasing in the x direction,

$$\varphi(x) = \left(\frac{2\pi\mu_0 t_d}{\Phi_0} H_{ext}\right) x + \varphi_0 = kx + \varphi_0. \tag{10}$$

Accordingly, equation (8) becomes

$$I_s(H) = \int_{-\mathcal{L}/2}^{\mathcal{L}/2} i_c(x)\sin(kx+\varphi_0)dx = \mathrm{Im}\left\{\int_{-\infty}^{\infty} i_c(x)e^{i(\varphi_0+kx)}dx\right\} = \mathrm{Im}\left\{e^{i\varphi_0}\int_{-\infty}^{\infty} i_c(x)e^{ikx}dx\right\}. \quad (11)$$

The Josephson critical current is the amplitude of the last integral, that is

$$I_s^m(H) = \left|\int_{-\infty}^{\infty} i_c(x)e^{ikx}dx\right|, \quad (12)$$

independent of any phase factor φ_0.

By assuming a uniform supercurrent area density within the junction, i.e., $J_c(x,y) \equiv J_c$, for $0 \le x \le \mathcal{L}$ and $0 \le y \le \mathcal{W}$, and zero elsewhere, we obtain $i_c(x) \equiv i_c = J_c\,\mathcal{W}$, according to equation (7). Therefore, equation (12) becomes

$$I_s^m(H) = \left|i_c\int_{-\mathcal{L}/2}^{\mathcal{L}/2} e^{ikx}dx\right| = i_c\left|\int_{-\mathcal{L}/2}^{\mathcal{L}/2}\cos(kx)dx\right| = \frac{I_c}{\mathcal{L}}\left|\int_{-\mathcal{L}/2}^{\mathcal{L}/2}\cos(kx)dx\right|, \quad (13)$$

and finally

$$\frac{I_s^m(\Phi)}{I_c} = \left|\frac{\sin\frac{\pi\Phi}{\Phi_0}}{\frac{\pi\Phi}{\Phi_0}}\right|, \quad (14)$$

where $k = \frac{2\pi\mu_0 t_d}{\Phi_0}H_{ext}$, Φ is the magnetic flux through the effective magnetic area ($t_d\,\mathcal{L}$), and $I_c = i_c\mathcal{L} = J_c\mathcal{L}\mathcal{W} = J_c\mathcal{A}$.

The long junction case markedly differs with respect to the short case, since both the penetrating external field and the self-field generated by the Josephson current have to be considered, so that $\varphi(x)$ nonlinearly changes along the junction according to Eqs. (1)-(2). Therefore, in normalized units, the maximum value of the Josephson current can be written as

$$\frac{I_s^m(t)}{I_c} = \frac{1}{L}\left|\int_0^L dx\cos\varphi(x,t)\right|. \quad (15)$$

It only remains to include in equation (15) the proper phase difference $\varphi(x,t)$ for a driven long JJ given by solving Eqs. (1)-(2). The magnetic field dependence of I_s^m results in "Fraunhofer-like" diffraction patterns [8, 9, 5]. While in the short junction limit [2], different diffraction *lobes* are well separated, here we observe the overlapping of the lobes. The transitions between these lobes are usually discontinuous. These patterns can be explained in terms of solitons entering the JJ.

Each lobe corresponds to a state with a fixed number of solitons. When the magnetic field increases, the configuration with more solitons is energetically favorable and, thus, the system jumps from a metastable state to a more stable state with more solitons. In the region of H values in which the diffraction lobes overlap, several solutions with different number of solitons may co-exist [8, 9]. Therefore, the system stays in the present configuration until the following one is energetically more stable.

To further explore the behavior of a magnetically driven LJJ, we have implemented a double-swept drive. The forward, i.e., with H increasing, and the backward, i.e., with H decreasing, patterns are significantly different. For a given value of the magnetic field, the critical currents in the backward and forward evolutions differ and the system is found in a different diffraction lobe. We can associate the forward and backward stable states (at fixed H) with a different number of solitons in the junction. Interestingly, the overall effect is a hysteric behavior in the critical current.

The diffraction patterns of the Josephson critical current, as the driving field is first increased (forward plot) and then reduced (backward plot), are shown in Fig. 2 for several JJ normalized lengths.

Forward-backward differences in the hysteretic behavior of the critical current are strongly evident for $|H| \lesssim H_c = 2$, see Fig. 2.

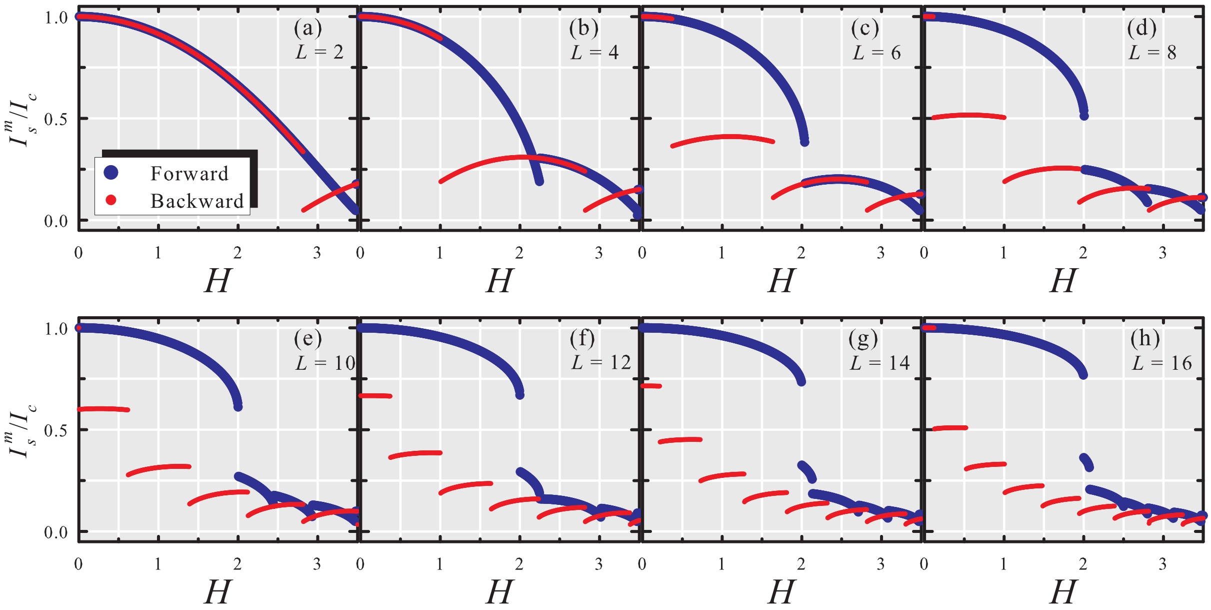

Figure 2: Forward and backward normalized critical currents I_s^m/I_c as a function of the magnetic field H setting the damping parameter $\alpha = 0.24$ for several JJ length $L = 2, 4, 6, 8, 10, 12, 14,$ and 16 [panels (a), (b), (c), (d), (e), (f), (g), and (h) respectively]. Specifically, H is swept first forward from H=0 and then backward. The legend in panel (a) refers to all panels.

The differences between forward and backward solutions suggest applications of this system as *multi-state memories*, in which each state is clearly indicated by drastic suppressions of the critical current I_s^b with respect to I_s^f. To quantify the LJJ memory-device performance we make use of a figure of merit defined by the difference between the critical currents,

$$\delta I_i = \frac{\left| I_s^f(H_i) - I_s^b(H_i) \right|}{I_c}, \tag{16}$$

where H_i is the magnetic field at the midpoint of the i-th backward diffraction lobe. For large δI_i one can safely distinguish distinct memory states (MSs), namely, the current states. For instance, by focusing on the panels c,d, and e of Fig. 2, we observe that, in the range $H \in [0 - H_c]$,

- for $L = 6$, only one MS is clearly available, with a current difference $\delta I_1(H_1 \simeq 1) \sim 0.5$, see Fig. 2c;
- for $L = 8$, two MSs can be defined, with $\delta I_1(H_1 \simeq 0.5) \sim 0.5$ and $\delta I_2(H_2 \simeq 1.5) \sim 0.6$, see Fig. 2d;
- for $L = 10$, three MSs can be defined, with $\delta I_1(H_1 \simeq 0.32) \sim 0.4$, $\delta I_2(H_2 \simeq 1) \sim 0.6$, and $\delta I_3(H_3 \simeq 1.75) \sim 0.6$, see Fig. 2e.

Finally, junctions with different lengths are characterized by different numbers of distinct available MSs, each of them corresponding to a specific amount of solitons arranged along the junction.

Moreover, for a fixed effective junction length $\mathcal{L}$, the normalized length $L(T) = \mathcal{L}/\lambda_{_J}(T)$ and, therefore, the amount of MSs of the memdevice can be controlled by changing the temperature T of the system.

3 Thermal effects

We observe that the plasma frequency $\omega_p(T) = \sqrt{\frac{2\pi}{\Phi_0}\frac{I_c(T)}{C}}$, the damping parameter $\alpha(T) = \frac{1}{\omega_p(T)RC}$, the Josephson penetration depth $\lambda_{_J}(T) = \sqrt{\frac{\Phi_0}{2\pi\mu_0}\frac{1}{t_d(T)J_c(T)}}$, and the normalized length $L(T) = \mathcal{L}/\lambda_{_J}(T)$ vary by changing the temperature.

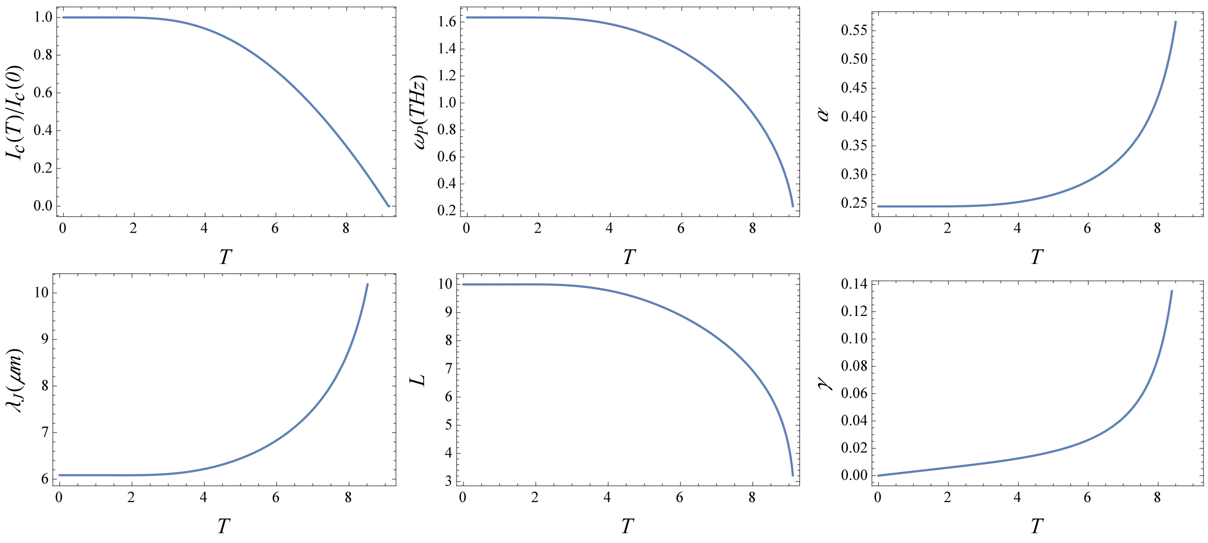

Figure 3: Normalized Josephson critical current I_c, plasma frequency ω_p, damping parameter α, Josephson penetration depth λ_J, normalized length L, and thermal noise amplitude γ as a function of the temperature, for $T_c = 9.2K$.

The temperature of the bath influences also the dynamics of the junction. In order to take into account the thermal fluctuations on the phase dynamics, a noise current i_T has to be included into the perturbed SG equation

$$\frac{\partial^2 \varphi}{\partial t^2} + \alpha(T)\frac{\partial \varphi}{\partial t} - \frac{\partial^2 \varphi}{\partial x^2} = -\sin(\varphi) + i_T\,(x,t)\,. \tag{17}$$

The normalized thermal current $i_T\,(x,t)$ is characterized by the well-known statistical properties of a Gaussian random process

$$\langle i_T\,(x,t)\rangle = 0 \qquad\qquad \langle i_T\,(x,t)\, i_T\,(x',t')\rangle = 2\gamma(T)\delta\,(x-x')\,\delta\,(t-t')\,. \tag{18}$$

For a LJJ, the thermal noise amplitude reads [10]

$$\gamma(T) = \frac{2\pi}{\Phi_0}L(T)\alpha(T)\frac{k_bT}{I_c(T)}. \tag{19}$$

The behaviors of the normalized Josephson critical current I_c, the plasma frequency ω_p, the damping parameter α, the Josephson penetration depth λ_J, the normalized length L, and the thermal noise amplitude γ as a function of the temperature, for $T_c = 9.2K$, are shown in Fig. 3.

Moreover, the values of the thermal noise amplitude, the damping parameter, and the plasma frequency in correspondence of few specific temperatures (used to obtain the results shown in the manuscript) are listed in Table 1.

T[K]	$\gamma(T)$	$\alpha(T)$	$\omega_p(T)$[THz]
0.02	0.00006	0.244	1.634
0.3	0.00088	0.244	1.634
1.2	0.00351	0.244	1.634
4.2	0.01346	0.254	1.573
$0.75 \times T_c = 6.9$	0.03997	0.328	1.221
$0.9 \times T_c = 8.3$	0.11660	0.494	0.810

Table 1: Thermal noise amplitudes, damping parameters, and plasma frequencies in correspondence of few specific temperatures used to obtain the results shown in the manuscript.

4 The frequency response

We explore the effects of variations of the driving frequency on the behavior of our device. However, our system is an example of a memory that benefits from, and properly work only in, the presence of noise. To discuss this point, we compare results obtained in both the deterministic and stochastic approaches, by taking into account several temperatures.

To quantify the LJJ-based memory performances as the driving frequency is changed, we use the distances δI_i defined in equation (16). Specifically, Fig. 4a shows the midpoint values H_i of the backward diffraction lobes within the field range $H \in [0-2]$, and the distances δI_i $(i = 1, 2, 3)$ for these fields, for a junction with $L = 10$.

First, we analyse the device performance in absence of thermal noise. The behavior of δI_i $(i = 1, 2, 3)$ as the driving frequency ω_H is changed is shown in Fig. 4b for the deterministic case, i.e., no noise source is considered in the model. For the sake of clarity, we define in Fig. 4b two threshold frequencies, ω_1 and ω_2, and examine the results in different frequency ranges:

- for $\omega_H \lesssim \omega_1$ the distances δI_i approach constant values, inasmuch steady diffraction patterns are obtained. For these frequencies, the logic states are definitively robust against frequency variations;
- in the range $\omega_H \in [\omega_1 - \omega_2]$ the values of δI_i, and accordingly the amount of the logic states, significantly deviate from the steady ones;
- for $\omega_H \gtrsim \omega_2$ the system is not able to respond to extremely high driving oscillations, so that the backward patterns are highly disordered, despite the fact that $\delta I_i \to 1$, and therefore the logical states cannot be safely distinguished.

As discussed above, realistic devices are subject to thermal noise. The addition of the thermal noise has the effect to stabilise the dynamics and, therefore, to access to higher driving frequencies.

In the noisy approach, the distances

$$\delta\overline{I_i} = \frac{\left|\overline{I_s^f}(H_i) - \overline{I_s^b}(H_i)\right|}{I_c}, \tag{20}$$

are taken into account. The behaviors of $\delta\overline{I_i}$ $(i = 1, 2, 3)$ as a function of ω_H for $T = \{0.02, 0.3, 1.2, 4.2\}$K are shown in panels c, d, e, and f of Fig. 4, respectively. The quantities $\overline{I_s^f}/I_c$ and $\overline{I_s^b}/I_c$ are computed by averaging over the total number of numerical realizations, $N_{exp} = 100$, the normalized critical currents as the magnetic field H is swept forward and backward, respectively, when the thermal fluctuations are included in the SG model.

For $T = 0.02$K, 0.3K, and 1.2K (see Figs. 4c, d, and e, respectively) the values of $\delta\overline{I_i}$ are roughly constant and the logic states of the device are definitively stable up to $\omega_H \sim 0.1$GHz. Conversely, for higher frequencies, the inability of the system to adjust its state to rapid changes in the magnetic bias comes to light.

For $T = 4.2$K, i.e., the liquid helium temperature, the frequency behavior significantly changes, see Fig. 4f. In fact, $\delta\overline{I_i}$ $(i = 1, 2, 3)$ approach the values obtained for lower temperatures only for $\omega_H \sim 0.1$GHz. Conversely, for lower frequencies the thermal fluctuations have enough time to guide the evolution of the system, so that the state of the system is set by noise-induced transitions. Therefore, the backward and forward patterns tend to superimpose and $\delta\overline{I_i} \to 0$.

Moreover, we verify if the system is able to provide information-storage times longer than any practical reading times, so that it works as a *non-volatile memory* [11]. To this end, we show in Fig. 5 the normalized critical currents I_s^m/I_c and driving field H as a function of the normalized time t for the states defined in Fig. 4a, for $T = 1.2K$. Specifically, results in panels a, b, and c of Fig. 5 are obtained by freezing the magnetic field to $H(t \geq t_i) = H_i$ with $H_i = 0.32, 1.0,$ and 1.75, respectively, t_i being the time for the magnetic field to reach the value H_i during the backward sweep. In spite of the thermal fluctuations, as the magnetic field is set to $H(t) = H_i$, the critical current is roughly constant, i.e., $I_s^m(t \geq t_i) \sim I_s^m(t_i)$, so that steady logic states are established.

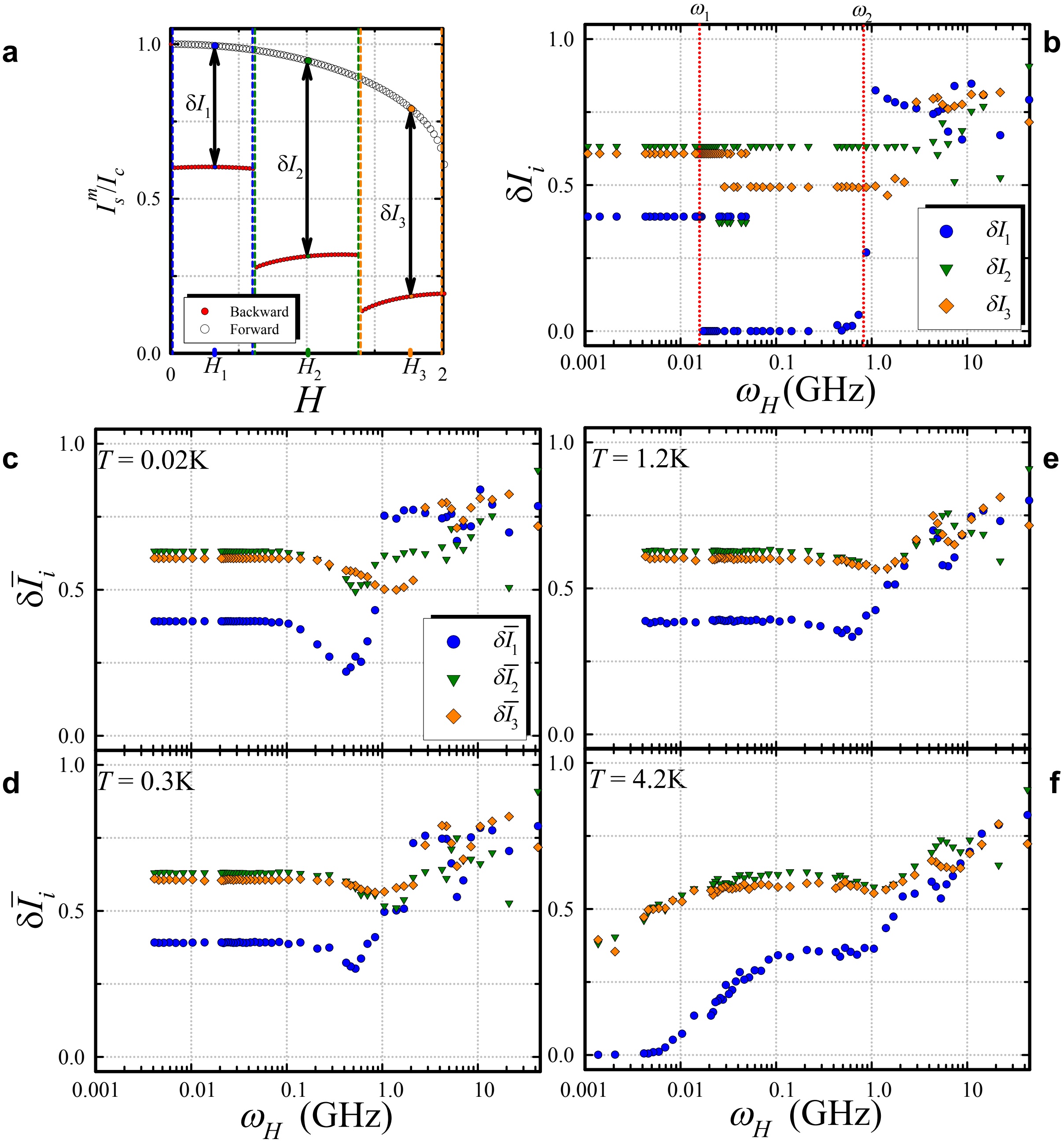

Figure 4: **a**, Forward and backward diffraction patterns for $H \in [0-2]$ and $L = 10$. The backward pattern is composed by three lobes in the place of the large lobe of the forward one. The value of the magnetic field H_i in the center of each lobe and the differences δI_i $(i = 1, 2, 3)$, see equation (16), for $H \equiv H_i$ are also shown. The values of δI_i are used to check the behavior of the logic states of the LJJ-based memory against frequency variations. **b**, δI_i $(i = 1, 2, 3)$ as a function of the driving frequency $\omega_H = \omega_p/T_H$ in absence of thermal noise. As ω_H reduces, the diffraction patterns tend to become steady and δI_i approach constant values. Specifically, by defining two threshold values, ω_1 and ω_2, the behavior of the device in different ranges of frequencies can be discussed: *i*) for $\omega_H \gtrsim \omega_2$ the system is not able to respond to extremely high driving frequency oscillations; *ii*) in the range $\omega_H \in [\omega_1 - \omega_2]$ the memory cannot safely provide three logic states ; *iii*) for $\omega_H \lesssim \omega_1$ the distances δI_i approach constant values, and, in spite of frequency variations, the system provides three distinct states. **c**, **d**, **e**, and **f**, distances $\delta\overline{I_i}$ $(i = 1, 2, 3)$, see equation (20), as a function of ω_H for $T = \{0.02, 0.3, 1.2, 4.2\}$K, respectively. The legend in panel **c** refers to all these panels.

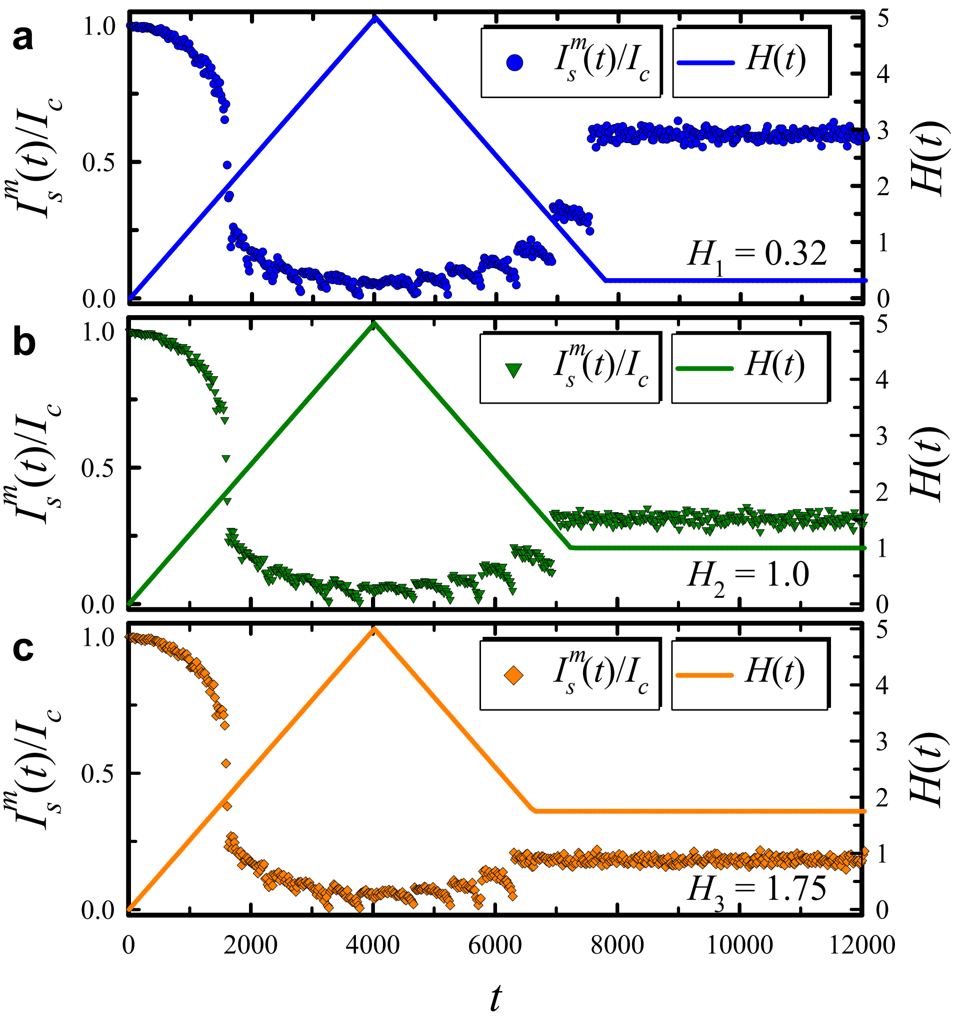

Figure 5: Normalized critical current I_s^m/I_c (left ordinate scale, full symbols) and driving field H (right ordinate scale, solid lines) as a function of the time t, normalized to ω_p, for the MSs defined in Fig. 4a for $L = 10$ and $H_i = 0.32, 1.0, 1.75$ with $i = 1, 2, 3$ (panels a, b, and c, respectively). The magnetic field value H_i is chosen in the midpoint of the i-th backward diffraction lobe, so that the robustness against small field fluctuations is ensured. These graphs are obtained by setting $H(t \geq t_i) = H_i$, where t_i is the time for the magnetic field $H(t)$ to reach the value H_i during the backward sweep. The diffraction patterns are computed for $T = 1.2K$. In spite of the thermal fluctuations, as the magnetic field is set to $H(t) = H_i$, the δI_i values are roughly constant in time, i.e., $I_s^m(t \geq t_i) \sim I_s^m(t_i)$. These results are obtained by setting $\Delta t_H = 4$, $\Delta H = 0.005$, and $H_{max} = 5$, so that the non-normalized driving frequency is $\omega_H = \omega_p/T_H \simeq 0.1$GHz, where $\omega_p(T = 1.2\text{K}) \simeq 1.634$THz (see Table 1).

5 Memory devices and the response function

The properties of a memory-element (*memelement* for short) depend on the state and the history of the system [12]. Specifically, in *ideal memristors*, *memcapacitors* and *meminductors* [12]

- (ideal memristor) the resistance depends only on the charge that flows in the system (or on the history of the voltage);
- (ideal memcapacitor) the capacitance depends only on the history of the charge stored on its plates (or the history of the voltage across it);
- (ideal meminductor) the inductance only depends on the history of the current that flows through

it (or the history of the flux).

These definitions can be generalized by invoking a general non-linear, memory-dependent response function g [13]

$$\begin{aligned} y(t) &= g(x,u,t)u(t) \\ \dot{x} &= f(x,u,t) \end{aligned} \tag{21}$$

where

- $g(x,u,t)$ is the response function;
- $u(t)$ is the input signal;
- $y(t)$ is the output signal;
- $f(x,u,t)$ is a vector function of internal state variables;
- x is a vector of internal state variables.

Generally, in real systems ideal memdevices are usually rare, so that the relation between current and voltage defines a *memristive system* (i.e., the resistance depends on both the charge and other internal variables of the system), while the relation between charge and voltage specifies a *memcapacitive system*, and the flux-current relation gives rise to a *meminductive system* [12].

A distinctive signature of memory devices is the presence of a *hysteresis loop* in the behavior of the output $y(t)$ and/or the response function $g(t)$ as a function of the input $u(t)$ [12]. The features of the hysteresis loop depend on the properties of both the system and the input $u(t)$, such as its amplitude and frequency. Hysteresis loops can be *pinched*, when the loop passes through the origin (y is zero whenever u is zero and vice versa). Moreover, a pinched hysteresis can be self-crossing [12], i.e., with the crossing between opposite direction branches of the loop, or not self-crossing.

In contrast with usual memelements defined by Eqs. (21), the behavior of our LJJ-based memory-device is not directly stated in the form of a relation between $I_s^m(t)$ and $H(t)$ through a response function $g(\varphi, H, t)$. In other words, our memdevice is not described by a current-field expression such as $I_s^m(t) = g(\varphi, H, t)H(t)$. In fact, in normalized units the critical current reads

$$\frac{I_s^m(t)}{I_c} = \frac{1}{L}\left|\int_0^L dx \cos\varphi(x,t)\right|. \tag{22}$$

The internal state variable of this field-controlled memelement is the phase difference $\varphi(x,t)$, whose dynamics is ruled by Eqs. (1)-(2).

We observe that a relation including a response functional comes to light by first-order expanding the $\cos\varphi(x,t)$ term in equation (22) around the junction edge $x=0$, that is by ignoring the non-linearity of the problem,

$$\cos\varphi(x,t) \sim_{x=0} \cos\varphi(0,t) - \sin\varphi(0,t) \left.\frac{\mathrm{d}\varphi(x,t)}{\mathrm{d}x}\right|_0 x. \tag{23}$$

Therefore, equation (22) becomes

$$\frac{I_s^m(t)}{I_c} = \left|\frac{1}{L}\int_0^L \cos\varphi(x,t)dx\right| \sim \left|\frac{1}{L}\int_0^L \cos\varphi(0,t)dx - \frac{1}{L}\int_0^L \left[\sin\varphi(0,t) \left.\frac{\mathrm{d}\varphi(x,t)}{\mathrm{d}x}\right|_0 x\right] dx\right|. \tag{24}$$

According to equation (2), the previous equation reads

$$\frac{I_s^m(t)}{I_c} \sim \left|\frac{1}{L}\cos\varphi(0,t)L - \frac{\sin\varphi(0,t)}{L}H(t)\int_0^L xdx\right| \sim \left|\cos\varphi(0,t) + F(\varphi,H,t)H(t)\right|. \tag{25}$$

Here, we have defined the functional (i.e. response functional) $F(\varphi, H, t)$

$$F(\varphi,H,t) = -\sin\varphi_{_H}(0,t)\frac{L}{2}, \tag{26}$$

where the field-dependence of the phase dynamics is stressed.

References

[1] Lomdahl, P. S., Soerensen, O. H. & Christiansen, P. L. Soliton excitations in Josephson tunnel junctions. *Phys. Rev. B* **25**, 5737–5748 (1982).

[2] Barone, A. & Paternò, G. *Physics and Applications of the Josephson Effect* (Wiley, New York, 1982).

[3] Valenti, D., Guarcello, C. & Spagnolo, B. Switching times in long-overlap Josephson junctions subject to thermal fluctuations and non-Gaussian noise sources. *Phys. Rev. B* **89**, 214510 (2014).

[4] Guarcello, C., Valenti, D., Carollo, A. & Spagnolo, B. Effects of Lévy noise on the dynamics of sine-Gordon solitons in long Josephson junctions. *J. Stat. Mech.: Theory Exp.* **2016**, 054012 (2016).

[5] Guarcello, C., Giazotto, F. & Solinas, P. Coherent diffraction of thermal currents in long Josephson tunnel junctions. *Phys. Rev. B* **94**, 054522 (2016).

[6] Ustinov, A. V. Solitons in Josephson junctions. *Physica D* **123**, 315–329 (1998).

[7] McLaughlin, D. W. & Scott, A. C. Perturbation analysis of fluxon dynamics. *Phys. Rev. A* **18**, 1652–1680 (1978).

[8] Kuplevakhsky, S. V. & Glukhov, A. M. Static solitons of the sine-Gordon equation and equilibrium vortex structure in Josephson junctions. *Phys. Rev. B* **73**, 024513 (2006).

[9] Kuplevakhsky, S. V. & Glukhov, A. M. Exact analytical solution of a classical Josephson tunnel junction problem. *Low Temp. Phys.* **36**, 1012–1021 (2010).

[10] Castellano, M. G. *et al.* Thermally activated escape from the zero-voltage state in long Josephson junctions. *Phys. Rev. B* **54**, 15417–15428 (1996).

[11] Di Ventra, M. & Pershin, Y. V. The parallel approach. *Nat. Phys.* **9**, 200–202 (2013).

[12] Pershin, Y. V. & Di Ventra, M. Memory effects in complex materials and nanoscale systems. *Adv. Phys.* **60**, 145–227 (2011).

[13] Di Ventra, M., Pershin, Y. V. & Chua, L. O. Circuit elements with memory: Memristors, memcapacitors, and meminductors. *Proc. IEEE* **97**, 1717–1724 (2009).
